# Aberrant Methylation and Immune Microenvironment Are Associated With Overexpressed Fibronectin 1: A Diagnostic and Prognostic Target in Head and Neck Squamous Cell Carcinoma

**DOI:** 10.3389/fmolb.2021.753563

**Published:** 2021-10-20

**Authors:** Surui Sheng, Bing Guo, Zhentao Wang, Zhihua Zhang, Jieyu Zhou, Zirong Huo

**Affiliations:** ^1^ Shanghai Key Laboratory of Stomatology, Department of Oral and Maxillofacial—Head and Neck Oncology, Shanghai Ninth People’s Hospital, National Center for Stomatology, National Clinical Research Center for Oral Diseases, College of Stomatology, Shanghai Jiao Tong University School of Medicine, Shanghai, China; ^2^ Shanghai Key Laboratory of Tissue Engineering, Department of Plastic and Reconstructive Surgery, Shanghai Ninth People’s Hospital, Shanghai Jiaotong University School of Medicine, Shanghai, China; ^3^ Department of Otorhinolaryngology Head and Neck Surgery, Shanghai Ninth People’s Hospital, Shanghai Jiaotong University School of Medicine, Shanghai, China

**Keywords:** head and neck squamous cell carcinoma, fibronectin 1, prognosis, immune microenvironment, methylation

## Abstract

**Background:** Fibronectin 1 (FN1) is involved in cell adhesion and migration processes such as metastasis, wound healing, embryogenesis, blood coagulation, and host defense. However, the role of FN1 in the diagnosis and prognosis of head and neck squamous cell carcinoma (HNSCC) is far from understood.

**Methods:** FN1 expression profiles and clinical parameters from multiple HNSCC datasets were applied to evaluate the association between FN1 expression and HNSCC survival. We also identified FN1 expression in the mRNA and protein levels in 20 pairs of clinical samples by quantitative polymerase chain reaction (qPCR) and immunohistochemistry. Receiver operator characteristic (ROC) analysis was used to demonstrate the potential diagnostic value of FN1 in HNSCC. Aberrant methylation PPI networks were established using multiple bioinformatic tools based on TCGA database. The immune microenvironment and levels of immune checkpoints were investigated between groups with high and low FN1 expression.

**Results:** FN1 was significantly upregulated in HNSCC compared with para-carcinoma tissues on the basis of TCGA database and our clinical samples. Univariate and multivariate Cox regression analysis revealed that FN1 could be an independent indicator for prognosis of HNSCC. GO enrichment and KEGG pathway analysis demonstrated that cell adhesion, focal adhesion, and the PI3K-Akt signaling pathway might be involved in the potential mechanisms of FN1’s prognostic performance in HNSCC. Methylation of FN1 was also higher and closely associated with poorer survival in HNSCC. In addition, FN1 expression was positively correlated with three DNA methyltransferases (DNMT1, DNMT3A, and DNMT3B). Furthermore, FN1 was positively associated with CD4^+^ T cells, endothelial cells, macrophages, and NK cells and negatively correlated with CD8^+^ T cells

**Conclusion:** FN1 might be an independent prognostic biomarker for HNSCC patients. Hypermethylation, the aberrant proportions of immune cells, and the PI3K/Akt signaling pathway might be involved in the mechanism of FN1’s oncogene role in HNSCC.

## Introduction

Head and neck cancer is a worldwide deadly disease with an estimated annual incidence of more than 830,000 (Bray et al., 2018). The 5-year survival rate is only 40–50% and even lower among advanced cancer patients (Chauhan et al., 2015), although the examination and treatment has improved in recent decades. Head and neck squamous cell carcinoma (HNSCC) is the most common histological type, which accounts for about 4% of all new cancer diagnoses in the United States. It has been recognized as a highly heterogeneous malignant tumor, which can derive from various anatomical sites in the upper airway and digestive tract, including the mouth, pharynx, and larynx (Siegel et al., 2016). The heterogeneity renders clinical prognosis difficult to predict (Leemans et al., 2018). Clinical parameters such as TNM classification are commonly relied on for predicting the outcomes, which is far from accurate. Thus, there is an urgent necessity for a better understanding of the molecular alterations to make accurate early HNSCC diagnoses, improve the prognosis, and provide new therapeutic strategies.

Fibronectin 1 (FN1) is being increasingly considered as a part of tumor pathogenesis and contributes to various malignant behaviors in solid tumors (Kumra and Reinhardt, 2016; Kujawa et al., 2020). Some studies have revealed that overexpression of fibronectin in the pre-metastatic niche facilitates the adhesion of bone marrow–derived cells, which promotes tumor cell migration and cancer metastasis by providing support for tumor cells to escape from the primary site (Shinde et al., 2018; Kujawa et al., 2020). The deposition of fibronectin into the tumor extracellular matrix (ECM), followed by the formation of fibrin–fibronectin complexes, has been shown to facilitate tumor angiogenesis, proliferation, and metastasis (Malik et al., 2010). Therefore, the highly expressed fibronectin is a potential biomarker for the early diagnosis of malignant tumors and micrometastasis. Previous studies of HNSCC samples have revealed that FN1 is upregulated in the tumor stromal region and at the invasive front of the tumor (Kosmehl et al., 1999). However, whether FN1 expression could be used as a diagnostic or prognostic biomarker in HNSCC is not sufficiently understood.

In the present study, we assessed the expression and prognosis of FN1 in HNSCC for the first time. We identified FN1 as a reliable prognostic biomarker in HNSCC based on TCGA dataset and further validated its capability in our clinical samples. Furthermore, aberrant methylation, the immune microenvironment, and the protein–protein interaction (PPI) network were investigated, which could be interpreted with regard to the underlying mechanism of FN1’s role in HNSCC patients.

## Materials and Methods

### Microarray Data Collection and FN1 Filtering

We searched the gene expression profiles from the Gene Expression Omnibus (GEO, https://www.ncbi.nlm.nih.gov/) dataset (Modhukur et al., 2018) according to the following criteria: 1. the samples were verified by pathology as head and neck squamous cell carcinoma (including the oral cavity, oropharynx, nasopharynx, larynx, laryngeal pharynx, nasal cavity, and paranasal sinus). 2. The profiles included samples of squamous cell carcinoma and normal tissues of para-carcinoma. 3. The gene expression profiles of cell lines or experimental animals were excluded. As a result, two gene expression profiles, including GSE40290 and GSE55550, were selected. Then the database from The Cancer Genome Atlas (TCGA, https://portal.gdc.cancer.gov/) was included as well.

Differentially expressed genes (DEGs) were filtered with the threshold of adjusted *p* < 0.01 and log (fold change) > 2 or log (fold change) <−2. There were 339, 251, and 285 DEGs in TCGA, GSE40290, and GSE55550, respectively. Two-crossing or three-crossing genes were investigated and visualized using Bioinformatics and Evolutionary Genomics (http://bioinformatics.psb.ugent.be/webtools/Venn/) among different datasets. Further screening was performed according to the following criteria: 1) there was a significant difference in the expression of DEGs between HNSCC and para-carcinoma tissues in all of the three datasets. 2) Kaplan–Meier (KM) survival analysis revealed an association between DEG expression and the prognosis of HNSCC. *p* < 0.05 was set as the cut-off value for filtering.

### Expression Analysis of FN1

TCGA database was applied to investigate the FN1 expression at the mRNA level. A comprehensive investigation on the basis of the Human Protein Atlas (THPA, https://www.proteinatlas.org/) was performed to evaluate the protein expression level of FN1. Twenty patients who were diagnosed with HNSCC were retrospectively collected in Shanghai Ninth People’s Hospital Affiliated to Shanghai Jiaotong University School of Medicine from April 2021 to June 2021, which was performed in accordance with the ethical principles described in the Declaration of Helsinki and approved by the bioethics committee of Shanghai Ninth People’s Hospital Affiliated to Shanghai Jiaotong University School of Medicine (approval no. SH9H-2021-T300-1). Twenty pairs of samples were classified as the primary tumor and the para-carcinoma group. RNA was extracted and reversed to cDNA. Quantitative polymerase chain reaction (qPCR) was performed to explore the mRNA levels of FN1. The following primers were used in this study. FN1: forward, CAA​ATG​GTT​CAG​CCC​CAG​TCC, reverse, GTC​CGC​TCC​CAC​TGT​TGA​TTT​ATC. GAPDH: forward, CAT​GAG​AAG​TAT​GAC​AAC​AGC​CT, reverse, AGT​CCT​TCC​ACG​ATA​CCA​AAG​T. Immunohistochemistry (IHC) was performed as previously described (Huo et al., 2017). Sections were incubated with anti-FN1 antibody (1:600, 66042-1-lg, Proteintech Group, Rosemont, PA, USA) overnight at 4 °C. The density of FN1 expression was demonstrated by the percentage of staining cell infiltration.

### Prognostic and Diagnostic Analysis of FN1

Raw count of RNA-sequencing data and clinical information of HNSCC were downloaded from TCGA dataset. The one-way ANOVA test was executed to explore the differences in FN1 expression between HNSCC groups classified by multiple clinical characteristics on the basis of TCGA database, including age, gender, tumor stage, lymph node metastasis, TNM classification, and smoking habit. The Kaplan–Meier (KM) survival analysis was applied to evaluate the correlation between FN1 expression and 5-year overall survival (OS). To further explore whether FN1 expression was independent of the other clinical variables in HNSCC, univariate and multivariate Cox regression analysis was performed. The receiver operating characteristic (ROC) curve was used to assess the diagnostic ability of FN1 in HNSCC. Further comparison of the diagnostic capability between FN1 and the epidermal growth factor receptor (EGFR), another common biomarker in malignant tumors, was also applied. Then, ROC analysis was applied to predict the performance of FN1 expression and other clinical parameters, such as TNM classification, in 5-year OS outcomes of HNSCC patients.

### Methylation Analysis of FN1

Methylation of FN1 was detected using UALCAN (http://ualcan.path.uab.edu/) (Chandrashekar et al., 2017), a web tool which could evaluate epigenetic regulation of gene expression by promoter methylation based on TCGA dataset. Furthermore, MethSurv (https://biit.cs.ut.ee/methsurv/) (Edgar et al., 2002), which could provide univariable and multivariable survival analysis based on DNA methylation biomarkers using TCGA (Edgar et al., 2002), was performed to evaluate the association between the position distribution of methylation around CpG islands and the prognosis of the HNSCC patient. Moreover, we also explored the correlation between three DNA methyltransferases (DNMT1, DNMT3A, and DNMT1) and FN1 expression on the basis of TCGA database.

### Immune Cell Environment Analysis

To evaluate immune infiltrations in different HNSCC patient groups divided by FN1 expression levels, we applied unsupervised clustering and CIBERSORT algorithms such as immunedeconv, an R package which integrates six state-of-the-art algorithms, including MCP-counter, TIMER, CIBERSORT, xCell, quanTIseq, and EPIC (Finotello et al., 2019). The scores or proportions of tumor-infiltrating cells and the expression values of immune-checkpoint–relevant genes were compared among groups with different FN1 expression levels. The TIDE algorithm, a computational method to model two primary mechanisms of tumor immune evasion (Jiang et al., 2018), was performed to predict the different responses of FN1^high^ and FN1^low^ after the treatment of the immune checkpoint blockade (ICB).

### PPI Network and Pathway Analysis

The PPI network from STRING (https://string-db.org/) (Szklarczyk et al., 2015) was depicted and visualized using Cytoscape software v3.8.2 (Shannon et al., 2003). FN1-associated genes were presented *via* UALCAN (Chandrashekar et al., 2017). The molecular functions and signaling pathways involved in HNSCC were predicted using DAVID (Huang da et al., 2009), a web-based analysis platform for identifying enriched biological themes and visualizing these processes by Gene Ontology (GO) enrichment analysis and genes on Kyoto Encyclopedia of Genes and Genomes (KEGG) pathway maps.

### Statistical Analysis

All data were analyzed using the SPSS statistical package (version 24.0; SPSS Inc., Chicago, United States), and visualized using R software v4.0.3 (R Foundation for Statistical Computing, Vienna, Austria). The Student’s *t*-test was applied to compare the FN1 expression between HNSCC and para-carcinoma tissues. The Wilcoxon rank-sum test was performed to investigate the association between FN1 and clinical parameters. The correlation between FN1 expression and other genes was detected by Spearman’s correlation analysis. Univariate and multivariate Cox regression models were used to predict the prognostic ability of FN1. *p* < 0.05 was regarded as a difference with statistical significance.

## Results

### Gene Expression Profile Analysis and FN1 Filtering

**FIGURE 1:**
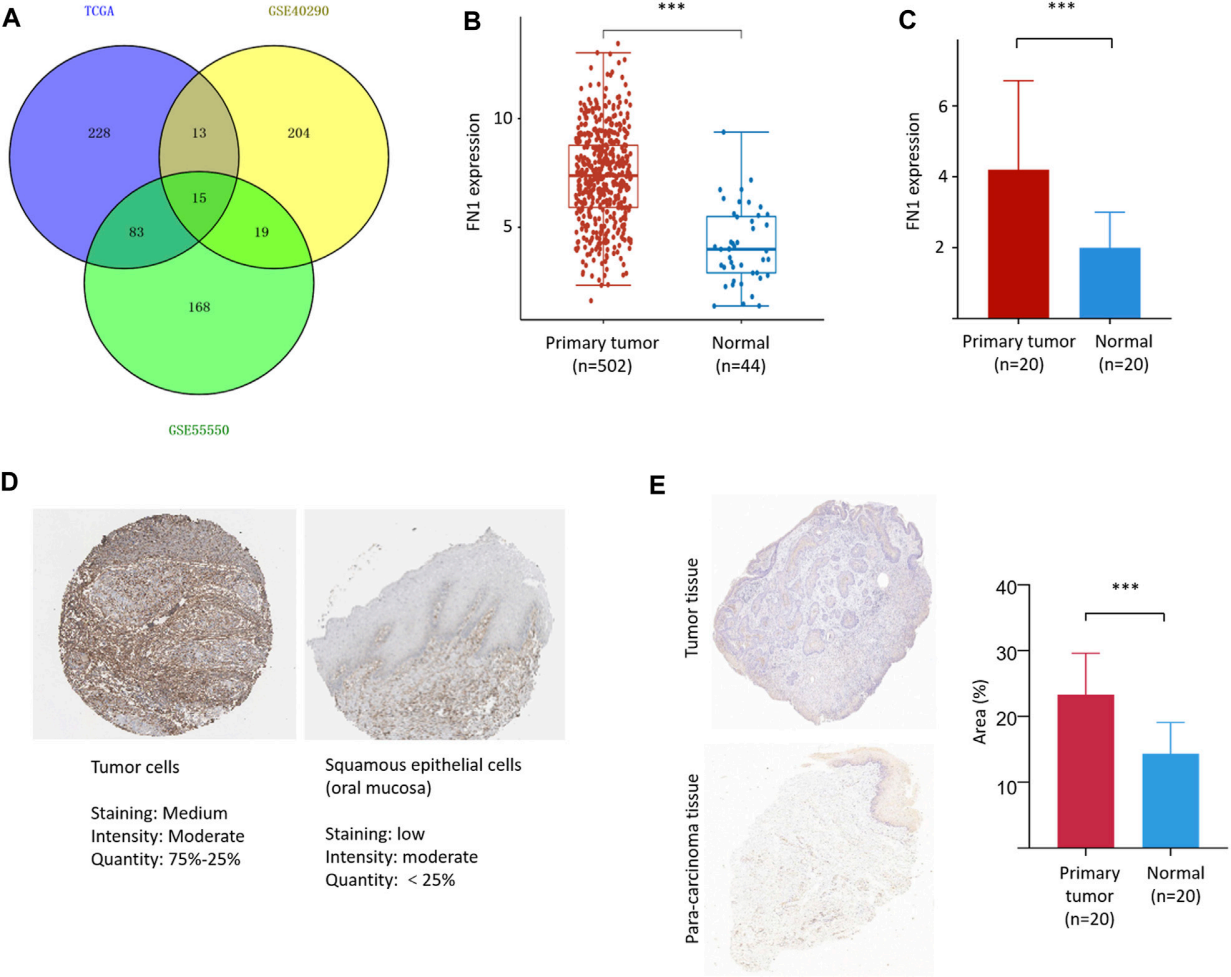
FN1 filtering and its expression in HNSCC. **(A)** A total of 15 three-crossing and 115 two-crossing DEGs were filtered among three public datasets. The mRNA level of FN1 was significantly higher in HNSCCs than in the normal para-carcinoma tissues from TCGA database **(B)** and our patients **(C)**. **(D)** The protein level of FN1 was much higher in HNSCC **(left)** than in the normal tissues of oral mucosa **(right)**, both from THPA. **(E)** Representative pictures of FN1 expression between different groups examined by IHC were shown **(left)** and verified by density value **(right)** and our patients.

**FIGURE 2:**
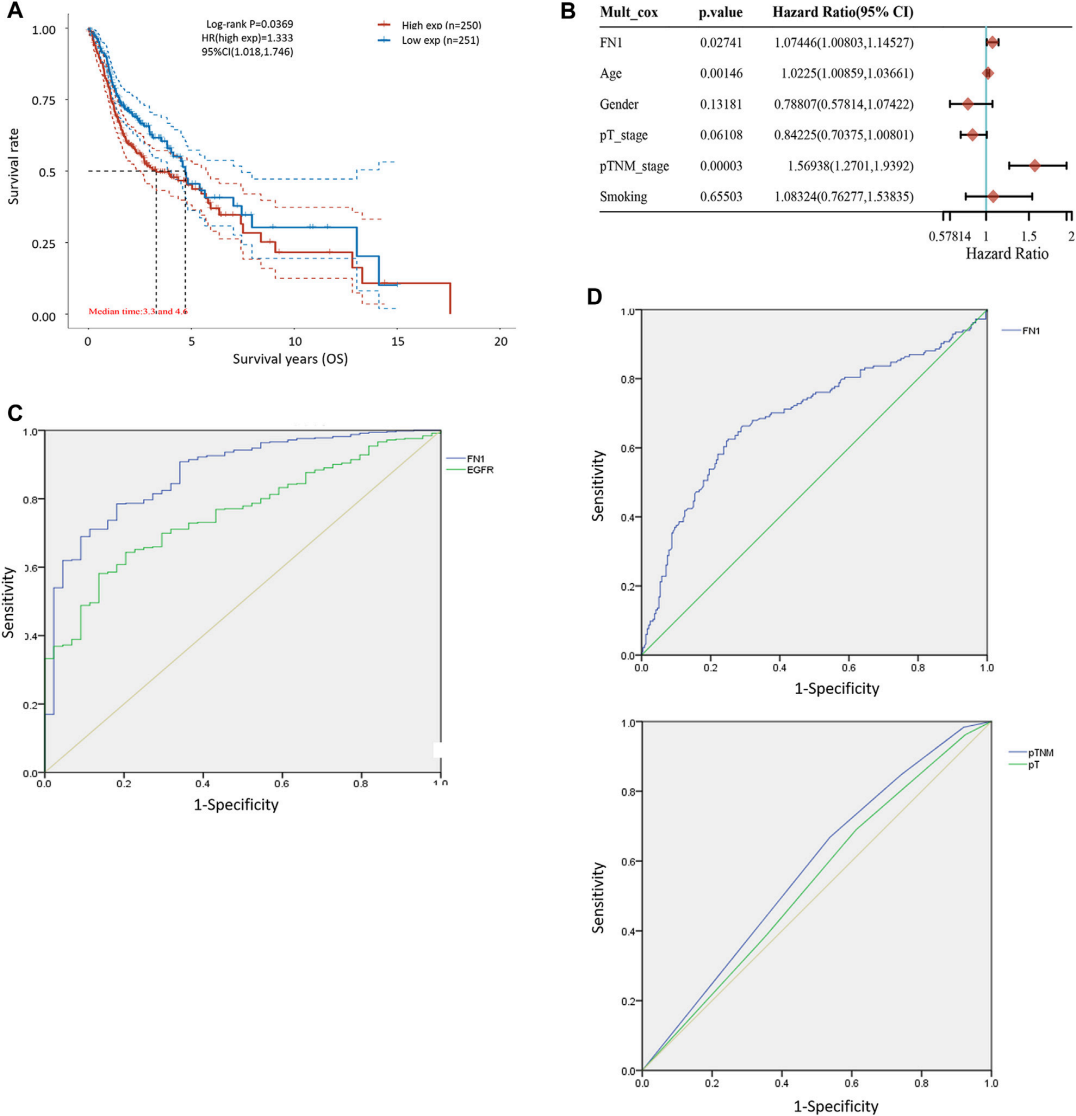
Diagnostic and prognostic capability of FN1 in HNSCC was examined using the ROC curve. **(A)** KM survival analysis showed that higher expression of FN1 was associated with worse prognosis of HNSCC patients. **(B)** Age, TNM classification, and FN1 expression were important prognostic factors for HNSCC patients examined by multivariate Cox analysis. **(C)** AUC of FN1 expression was higher than that of EGFR. **(D)** AUC of FN1 expression **(upper)** was higher than that of TNM classification or the tumor stage **(lower)**.

A total of 15 three-crossing and 115 two-crossing DEGs (13 between TCGA and GSE40290, 83 between TCGA and GSE55550, and 19 between GSE55550 and GSE40290) were filtered (Supplementary Table S1 and Figure 1A). Three DEGs (FN1, PLAU, and FAM3D) were filtered by KM survival analysis (Supplementary Table S2 and Figure 2A). We selected FN1 in the present study for its potential role in tumor angiogenesis and metastasis reported in previous studies (Malik et al., 2010; Kujawa et al., 2020).

### High Expression of FN1 in HNSCC

FN1 was significantly overexpressed in HNSCCs compared with normal para-carcinoma tissues on the basis of TCGA database (Figure 1B) and in our 20 matched pairs of clinical samples (Figure 1C). The protein expression level of FN1 was much higher in HNSCC than in the normal tissues of oral mucosa from THPA tool (Figure 1D). Similar results were found in our patients (Figure 1E). The clinical details of our patients are shown in Supplementary Table S3.

### FN1 Was a Predictor of HNSCC Prognosis

The associations between FN1 expression and some clinical parameters, including gender, age, tumor stage, lymph mode metastasis, TNM classification, and smoking status, were investigated. As shown in Table 1, there were significant differences among the four groups according to TNM classification (*p* = 0.027), and higher expression levels of FN1 were found in patients classified as stage IV than in the combined groups of the other three stages (*p* = 0.021). The results of univariate and multivariate Cox regression analysis showed that age, TNM classification, and FN1 expression were important prognostic factors for HNSCC patients (Table 2 and Figure 2B). Older patients and the patients with higher FN1 expression or a more advanced TNM stage were prone to having a worse survival outcome.

**TABLE 1:** Association between FN1 expression and clinical variables in HNSCC patients.

Clinical variable	Number	FN1 expression	*p* value
Gender			
Male	368	7.356 ± 2.123	0.590
Female	134	7.239 ± 2.161	
Age			
<60	221	7.346 ± 2.167	0.943
≥60	256	7.332 ± 21.123	
Tumor stage			
T1	34	6.805 ± 1.861	0.456
T2	144	7.318 ± 2.132	
T3	133	7.377 ± 2.221	
T4	177	7.445 ± 2.216	
Lymph metastasis			
N0	241	7.271 ± 2.102	0.650
N1	81	7.332 ± 2.049	
N2	136	7.481 ± 2.176	
TNM classification			
Ⅰ	25	7.472 ± 2.335	0.027^a^
Ⅱ	81	7.273 ± 1.949	
Ⅲ	90	6.734 ± 1.984	
Ⅳ	306	7.501 ± 2.180	
Smoking status			
Yes	381	7.367 ± 2.111	0.338
No	111	7.141 ± 2.206	

Complete data was unavailable in TCGA database.

a *p*<0.05.

**TABLE 2:** Univariate Cox regression analysis of prognostic factors of HNSCC.

Variable	OS		
	Hazard ratio	95% CI	*p* value
Gender (male/female)	0.764	(0.574, 1.018)	0.066
Age, year	1.021	(1.008, 1.034)	0.001^a^
Smoking status (yes/no)	1.089	(0.778, 1.525)	0.618
Tumor stage (T1/T2/T3/T4)	1.081	(0.940, 1.243)	0.276
TNM classification (Ⅰ/Ⅱ/Ⅲ/Ⅳ)	1.372	(1.162, 1.620)	<0.001^b^
FN1 expression	1.067	(1.000, 1.137)	0.049^c^

OS, overall survival; CI, confidence interval.

a *p* < 0.1.

b *p* < 0.001.

c *p*<0.05.

### Diagnostic and Prognostic Capability of FN1 in HNSCC

The receiver operator characteristic (ROC) curve was applied to examine the diagnostic ability of FN1 in HNSCC patients. The area under the curve (AUC) of FN1 expression was 0.875 (Figure 2C), higher than that of epidermal growth factor receptor (EGFR) expression (AUC = 0.756, *p* < 0.001), which has been regarded as a biomarker in multiple malignant carcinomas (Yip et al., 2017; Serilmez et al., 2019; Park et al., 2020). Furthermore, the predictive performance of FN1 expression in 5-year OS outcomes of HNSCC patients was analyzed. As shown in Figure 2D, the AUC of FN1 expression was 0.694 (Figure 2D upper), higher than that of TNM classification (AUC: 0.556) or that of the tumor stage (AUC: 0.543) (Figure 2D lower), with a sensitivity of 62.5% and a specificity of 75.0%. Taken together, FN1 might facilitate the diagnosis and OS prediction for HNSCC patients.

### Hypermethylation of FN1 and its Potential Prognostic Ability in HNSCC

**FIGURE 3:**
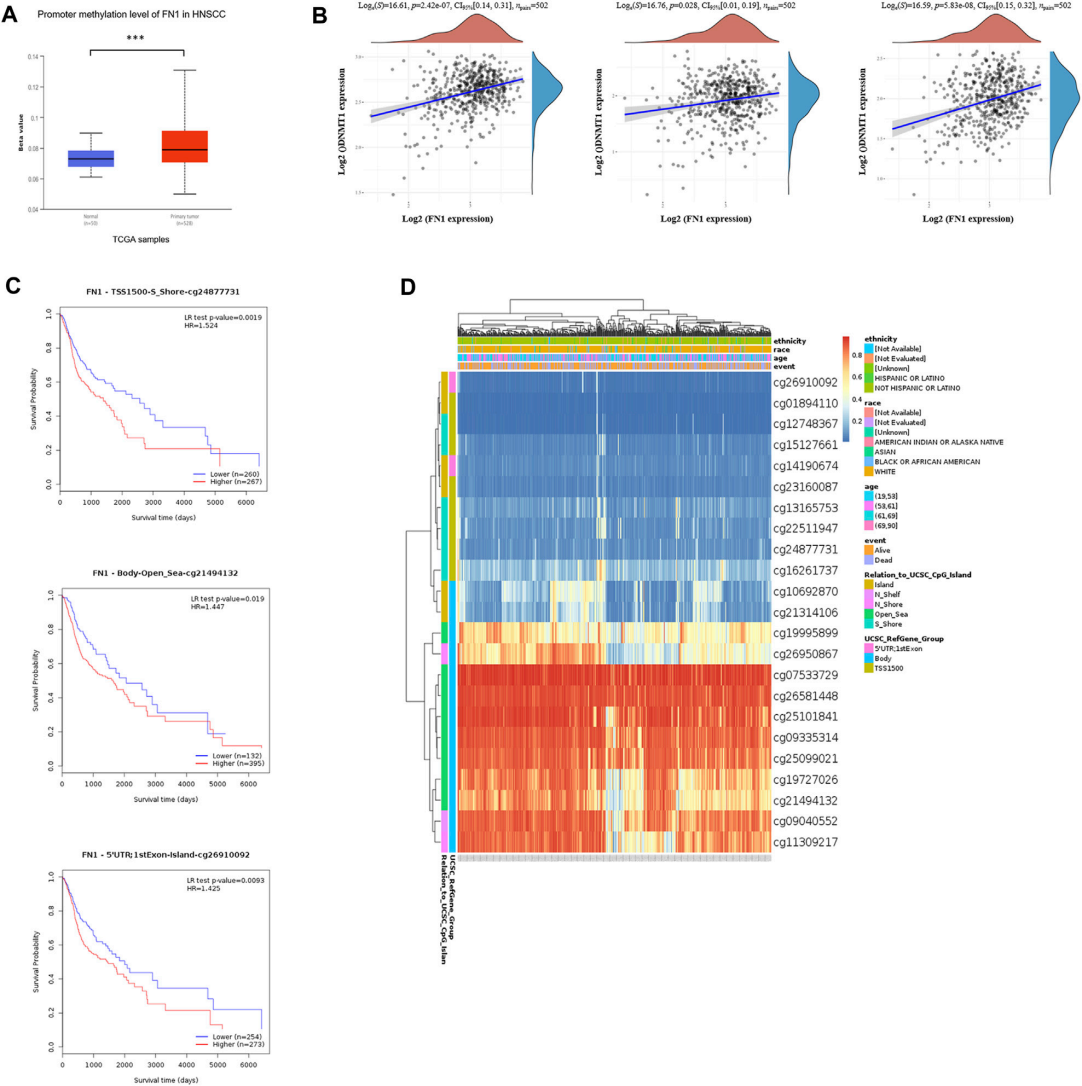
Hypermethylation of FN1 and its prognostic value in HNSCC. **(A)** FN1 was significantly hypomethylated in HNSCC tissues compared with the normal group. **(B)** FN1 expression was positively correlated with three crucial DNA methyltransferases’ (DNMT1, DNMT3A, and DNMT3B) levels. **(C)** Higher methylation of specific sites of FN1 predicted a shorter survival time compared to hypomethylation. **(D)** FN1-associated differently methylated regions were compared and visualized as the heatmap.

To further clarify the mechanism of FN1 overexpression in HNSCC, we investigated the methylation status *via* multiple tools. The analysis of UALCAN demonstrated that FN1 was significantly hypermethylated in HNSCC tissues compared with normal para-carcinoma tissues (Figure 3A). Besides, there was significant positive correlation between the expression levels of FN1 and three crucial DNA methyltransferases (DNMT1, DNMT3A, and DNMT3B) (Figure 3B). Survival analysis of different methylated regions was evaluated using the MethSurv tool. As shown in Figure 3C, higher methylation of FN1 predicted a shorter survival time than hypomethylation (TSS1500-S Shore-cg24877731, *p* = 0.002. Body-Open Sea-cg21494132, *p* = 0.019. 5′UTR; 1st Exon-Island-cg26910092, *p* = 0.009). FN1-associated differently methylated regions were compared and visualized as the heatmap (Figure 3D). The result revealed that more hypermethylated sites were located around the open sea regions, while more hypomethylated sites lied in CpG islands. In addition, relative position distributions in different locations of the gene were also depicted. More hypermethylated sites fell onto body regions, while more hypomethylated sites lied on TSS1500, 5′UTR, and 1st Exon regions.

### Immune Microenvironment of HNSCC With Different FN1 Expressions

**FIGURE 4:**
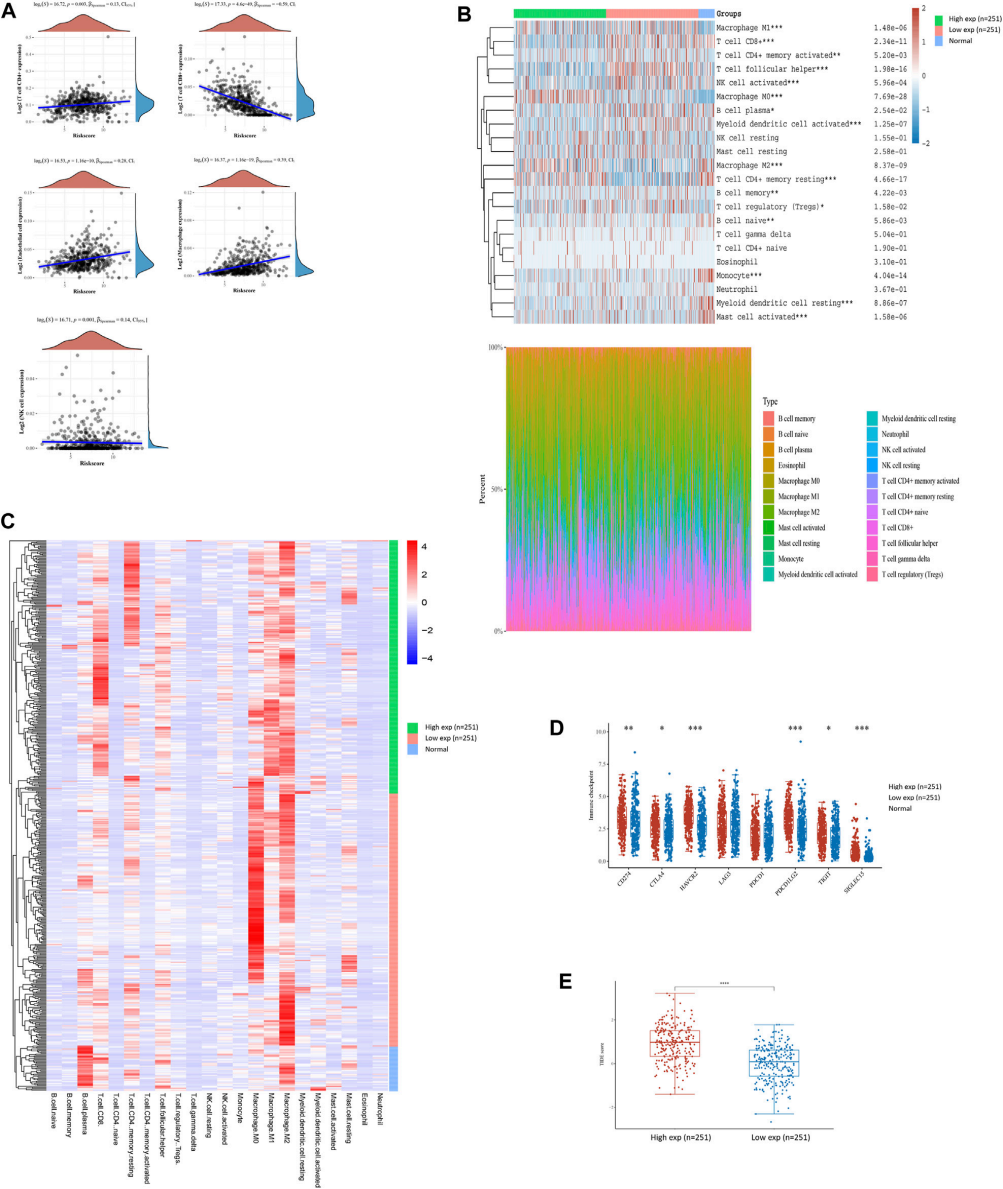
FN1-related tumor immune microenvironment of HNSCC. **(A)** FN1 expression was positively correlated with CD4^+^ T cells, endothelial cells, macrophages, and NK cells and negatively correlated with CD8^+^ T cells. Scores and percentage abundance of tumor-infiltrating cells in FN1^high^ and FN1^low^ as well as normal samples performed using the CIRBSORT algorithm **(B)** and unsupervised clustering **(C)**. **(D)** Immune-checkpoint–relevant genes were differentially expressed in FN1^high^ and FN1^low^ samples. **(E)** Patients with higher expression level of FN1 had a higher TIDE score.

To interpret the role of FN1 expression in the immune microenvironment of HNSCC, the proportions of tumor-infiltrating immune cells were investigated in the TCGA-HNSCC cohort. The results showed that FN1 expression was positively correlated with CD4^+^ T cells, endothelial cells, macrophages, and NK cells and negatively correlated with CD8^+^ T cells (Figure 4A). The scores and percentage abundance of tumor-infiltrating cells in FN1^high^ and FN1^low^ samples are presented as Figures 4B, C. Further analysis revealed that the immune-checkpoint–relevant genes were differentially expressed in HNSCC patients classified according to FN1 expression (Figure 4D). Potential ICB response was predicted using the TIDE algorithm. We found that the TIDE score was much higher in HNSCC patients with higher FN1 expression levels (Figure 4E), indicating a potential positive effect of immune checkpoint inhibitors in these patients.

### Network Establishment for FN1 Correlated Genes in HNSCC

**FIGURE 5:**
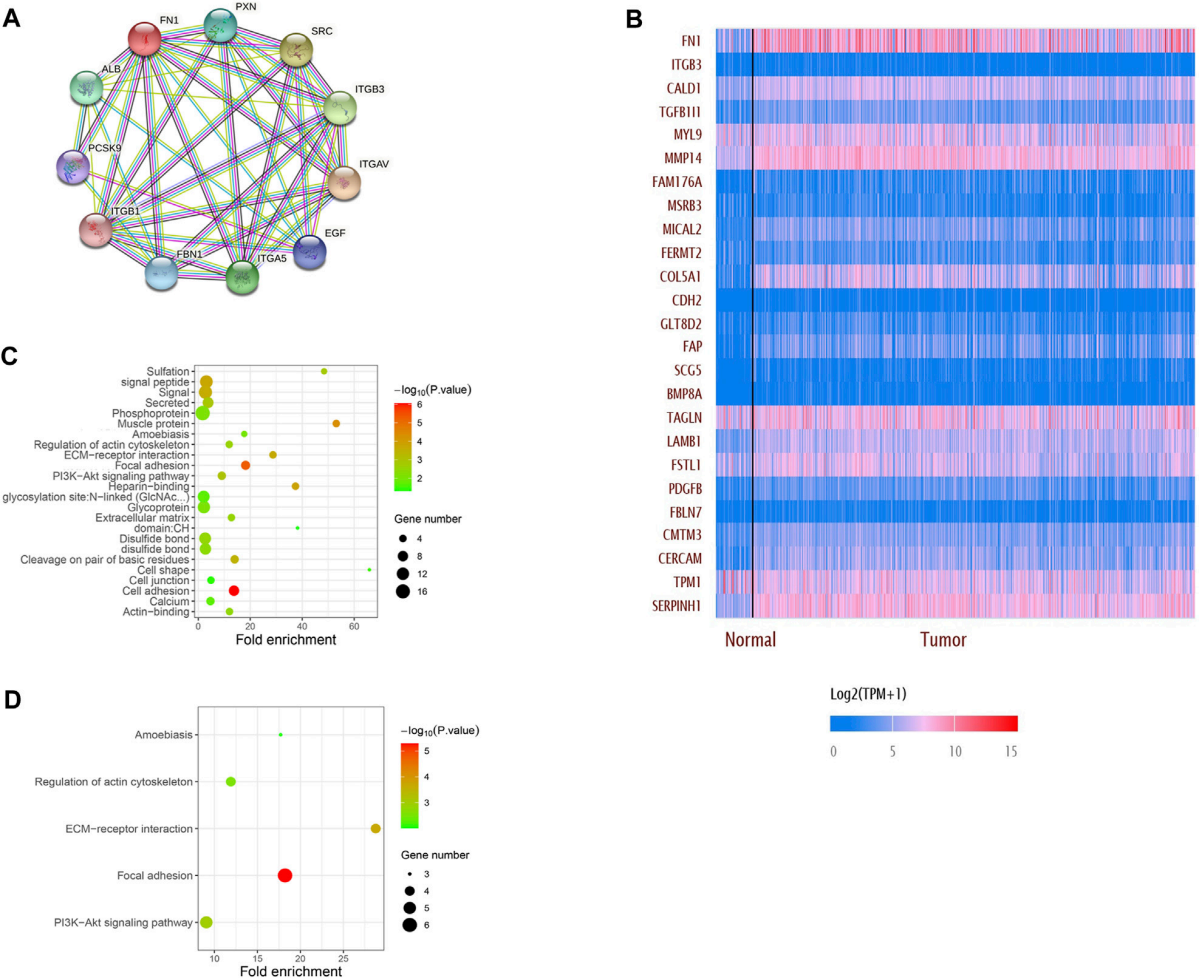
Network establishment for FN1-correlated genes in HNSCC. **(A)** PPI networks of FN1 interaction partners generated using STRING and Cytoscape. **(B)** Top 25 genes positively or negatively correlated with FN1 were shown in the heatmap. Major biological processes, cellular components, molecular functions and signaling pathways of FN1 biology by GO enrichment **(C)** and KEGG pathway analysis **(D)**.

The PPI network showed that FN1 was correlated with various genes. The top 10 related genes were predicted on STRING and visualized using Cytoscape software (Figure 5A). More genes positively or negatively correlated with FN1 were shown in a heatmap *via* UALCAN (Figure 5B). Gene Ontology (GO) analysis illuminated that the major biological processes (regulation of the actin cytoskeleton, cell junction, and cell adhesion), cellular components (ECM), and molecular functions (ECM–receptor interaction) might contribute to FN1-related biology. GO enrichment and KEGG pathway analysis showed that the P13K-Akt signaling pathway and focal adhesion were significantly enriched by the FN1 co-expressed genes (Figures 5C, D).

## Discussion

In the present study, we found that FN1 was upregulated in HNSCC, and its overexpression was correlated with a poorer prognosis. Furthermore, FN1 could be an independent prognostic factor for HNSCC patients, which was consistent with previous studies in other malignant tumors (Ruiz-Garcia et al., 2010; Kenny et al., 2014). As far as we know, this is the first study demonstrating that hypermethylation and an aberrant immune microenvironment might contribute to the overexpression of FN1 in HNSCC patients, which could provide a new perspective on the treatment of HNSCC.

Increased activation of FN1, a key component of the ECM, has been detected in metastasis and aggressiveness such as radio-resistance in various cancers (Van Obberghen-Schilling et al., 2011; Morita et al., 2015). While fibronectin (FN) is bound by multiple integrins at specific amino acid sequences, α5β1 integrin is selective for fibronectin (Mould et al., 1997). The binding of integrins to the ECM triggers integrin clustering and then promotes integrin-mediated intracellular signal transduction (Desgrosellier and Cheresh, 2010). At the same time, integrin receptors mediate matrix assembly of FN1 (Schwarzbauer and Sechler, 1999). Functionally, FN1 induces proliferation, adhesion, and invasion of tumor cells, contributing to the formation, adhesion, metastasis, and disaggregation of malignant tumors (Zand et al., 2003; Ritzenthaler et al., 2008; Mitra et al., 2011). On the other hand, it is confirmed that increased expression of FN1 is involved in the promotion of epithelial–mesenchymal transition (EMT), which has been implicated in tumor invasion and metastasis (Guarino et al., 2007; Margadant et al., 2012). EMT is characterized by disruption of intercellular contacts and enhancement of cell motility, facilitating malignant cells to invade surrounding tissues and subsequently enter the circulation, thereby allowing distant metastasis. In our present study, FN1 was found to be upregulated in HNSCC patients in TCGA cohorts and in our clinical paired samples. In addition, FN1 overexpression was associated with poorer outcomes of 5-year OS, which could be an independent factor for the prognosis of HNSCC. However, FN1 expression levels were not correlated with lymph node metastasis in our study, indicating that FN1 might induce metastasis of HNSCC through other processes, such as hematogenous metastasis.

DNA methylation, as an important part of epigenetics, is presented by the faithful cross–cell division transmission of the gene transcription memory (Lujambio et al., 2007). The hypermethylated promoter and enhancer regions tightly correlate with the transcriptional silence of both protein-coding and non-coding RNA genes, subsequently regulating gene expression, especially for tumor suppressor genes (Shi et al., 2016). Thus, exploring the DNA methylation state of the promoter, rather than the levels of the corresponding mRNAs or proteins, promises a better way for both early diagnosis and personalized therapy of cancer. In the current study, we analyzed methylation of the FN1 gene *via* multiple bioinformatic tools based on TCGA database. We found that FN1 was hypermethylated in HNSCC tissues. Consistent results showed that the FN1^High^ group co-occurred with higher expression of DNA methyltransferases (DNMT1, DNMT3A, and DMNT3B). Besides, the positive correlation between differently methylated sites and prognosis of HNSCC patients suggested that this epigenetic modification might be a potentially increased risk of HNSCC-related death. Although it has been known that inactivation of certain tumor-suppressor genes occurs as a consequence of hypermethylation within the promoter regions (Kulis and Esteller, 2010), our study observed a positive correlation between FN1 expression and methylation, which could be explained by the fact that the most hypermethylated sites were located in body regions of FN1. In line with our findings was a reported mechanism that positive correlations were enriched in the 3′UTR and body regions, and only 20% were located in TSSs (Gyorffy et al., 2016).

Growing evidence suggests that the innate immune cells (neutrophils, macrophages, innate lymphoid cells, dendritic cells, natural killer cells, and myeloid-derived suppressor cells) and adaptive immune cells (B cells and T cells) contribute to tumor progression when present in the tumor microenvironment (TME) (Gajewski et al., 2013). Therefore, the investigation of the TME in HNSCC may allow for improved therapeutics that target multiple components of the TME simultaneously, improving the outcomes for HNSCC patients. Furthermore, the administrations of immune checkpoint modulators (such as anti-CTLA4 and anti-PD antibodies) and adoptive immune cells (such as CAR-T) have exhibited unexpected antitumor effect in various cancers (Lei et al., 2020). Recent research has demonstrated that exposure to immune checkpoint inhibitors (ICIs) promotes tumor sensitivity to chemotherapy in HNSCC (Saleh et al., 2019). In this study, we found that FN1^High^ patients were prone to having higher levels of CD274 (PD-L1), CTLA4, and so on. Currently, the blockade of PD-1 signaling using the PD-1/PD-L1 antibody (such as pembrolizumab, nivolumab, and durvalumab) or the blockade of CTLA4 signaling using the CTLA4 antibody (such as ipilimumab and tremelimumab) has revealed the encouraging therapeutic effects in multiple cancers (Hodi et al., 2010; Topalian et al., 2012). Other checkpoint modulators, including agonistic antibodies (such as CD40 and GITR) and inhibitory antibodies (such as LAG-3), are still under clinical evaluation.

In our GO and KEGG pathway analysis, FN1 is involved in the synthesis of ECM components, and the PI3K-Akt pathway was enriched by FN1 co-expressed genes. Akt activation may be the downstream pathway of FN1 leading to tumor progression and poor prognosis in HNSCC. Previous reports have indicated that the PI3K/AKT/mTOR pathway is instrumental in FN transcription and alternative splicing, which modulates cell behavior (White et al., 2010). Matsuo et al. reported that the PI3K/Akt pathway was activated by FN with the aid of integrin αv-mediated a disintegrin and metalloprotease (ADAM) activity in hepatocellular carcinoma (Matsuo et al., 2006), providing us with a new understanding of the PI3K/Akt pathway in FN-related tumor progression.

Despite some merits of our study, limitations should be addressed. The analyses were performed based on the public datasets *via* multiple bioinformatic tools, which could provide a new perspective for our following research. Therefore, our findings need to be confirmed by more *in vitro* and *in vivo* experiments. In addition, the platforms applied in different cohorts are not the same, which may bring bias to the data analysis and difficulties for the deep integrated analysis.

In summary, we found that FN1 was overexpressed in HNSCC patients and upregulation of FN1 was correlated with a worse survival outcome. In addition, hypermethylation and an aberrant TME were strongly associated with FN1 overexpression *via* the PI3K/Akt signaling pathway. Therefore, FN1 could be considered as an independent diagnostic and prognostic biomarker in HNSCC.

## Data Availability

The datasets presented in this study can be found in online repositories. The names of the repository/repositories and accession number(s) can be found in the article/Supplementary Material.
