# Supplementary material for: Aberrant Methylation and Immune Microenvironment Are Associated With Overexpressed Fibronectin 1: A Diagnostic and Prognostic Target in Head and Neck Squamous Cell Carcinoma
Source: Front Mol Biosci. 2021 Oct 20;8:753563. doi: 10.3389/fmolb.2021.753563 (PMC8563786; doi:10.3389/fmolb.2021.753563)
Supplement: Supplementary file 1 [file Table1.DOCX]

**Supplementary Table 1.** Overlapping genes among three public datasets

| Names | Total | Elements |
| --- | --- | --- |
| GSE40290  GSE55550  TGGA | 15 | PLAU, KREMEN2, COL4A5, MMP3, POSTN, KRT4, IL24, CLU, FN1, COL4A1, VSIG2, COL10A1, PSCA, FAM3D, MMP1 |
| GSE40290  GSE55550 | 19 | MS4A1, SCGB1A1, CCL19, TCL1A, MSMB, CD19, RGS13, APLN, CCL7, CCL21, TNFAIP6, CR2, ATP12A, CD22, PTGS2, FCRLA, PRB4, CD1C, CRCT1 |
| GSE40290  TGGA | 13 | BPIFB1, CEACAM6, KLK13, CEP55, APOD, PIGR, FUT6, LTF, IL1A, COL11A1, ZG16B, COL1A2, ASPN |
| GSE55550  TGGA | 83 | TMPRSS11B, PLPP4, NXPH4, COL3A1, CCL11, MMP13, PADI1, HOXC13, TNC, MAB21L4, ENDOU, FAP, CLCA4, INHBA, COL1A1, TREM2, KRT13, GRIN2D, CEACAM5, PTHLH, PXDN, TMPRSS11E, IL36A, KRT16, CAPN14, EN1, MYOC, MAGEA6, THBS2, MAL, COL7A1, PLA2G7, CRNN, MFAP2, CXCL11, KRT17, SPINK5, TJP3, AQP5, CXCL10, FMO2, LAMB3, SERPINE1, CMYA5, FAM3B, COL5A2, LAMC2, LYPD2, SPRR3, PIP, COL6A3, SPP1, CA9, SH3BGRL2, MUC21, NEFL, SLURP1, TGM3, STC2, HTRA3, IL11, COL5A1, PRSS27, HPGD, RHCG, GREM1, SCEL, MMP10, PCSK9, KRT78, TFF3, CRISP3, MMP12, STATH, COL12A1, WDR66, CXCL17, MAGEA4, CSF2, TMPRSS11A, ATP6V0A4, MMP9, MMP11 |
| GSE40290 | 204 | DNAJC6, RBP5, RIT2, PTPRCAP, SPIB, SLA2, PARVG, LIPF, CXCL14, CD163, CD37, DPT, LILRA4, ARSF, SLC27A6, AQP9, UNC5A, CNR2, CAPS, MAGEB3, DMC1, PTGDS, KLHL1, MYO3A, PFKFB2, PIMREG, CRH, GPR183, ACSBG2, LRRK1, CPNE5, SLC8A1, CLDN16, DCP1A, TEKT1, OLFM4, SEPTIN1, CST7, SELL, GPRC5D, SPO11, FOXJ1, THBS4, CHST9, FXYD5, PF4, PLA2G2D, AQP3, GALP, RAB37, IL2, ALDH3A1, PLD4, CYSLTR2, BANF2, CCL8, NPEPL1, HCRTR2, TMEM190, CCDC17, SUPT5H, CD6, WNT8A, ST8SIA3, ADRA2A, GPSM3, GPR50, ARHGAP45, MPPED1, COL21A1, RHOH, TAAR2, VPREB3, EGFL6, HVCN1, LY86, PRSS21, RASGRP2, HTR3A, PDE7A, C4ORF45, ECRG4, IKZF3, FSCB, VXN, USP26, IL25, CD53, TTC29, IL31RA, PROM1, PTPN6, WFDC2, HOXA9, CD48, MNDA, FCRL5, NR5A1, MEGF10, ABCD2, CCDC81, ZSCAN31, SCN1A, COL17A1, CD180, IL17A, TSPAN1, MAP4K1, IL16, SMC5, DENND1C, TTC25, DEFB4A, EIF1AY, ACOXL, NCCRP1, ATP8B4, P2RX5, PPP1R16B, SNAP47, SIGLEC5, OR5P2, MEST, RSPH1, GMIP, TRAF3IP3, SPEF1, KLF2, TUBA1A, SELP, CLEC5A, SERPINB13, UPK1B, MS4A8, CD27, DEFB104A, EDDM3B, DYDC2, ADCY4, S1PR4, LRRC46, CCDC85A, GIMAP5, FAM81B, TFEB, SLPI, IRF8, F2RL3, HLADOB, WIPF1, ABI3BP, WDFY4, ODF2,L LAT2, LY9, S100A7A, LCN2, LST1, SH3GL2, VSIG4, RSPO3, TMOD2, ARHGAP24, RIPOR2, TNFSF18, MXRA5, C1QTNF2, ROPN1L, RAMP3, KCNJ5, EFCAB1, ZNF536, ZNF354A, CD72, FHL5, LRCH3, TIMD4, FCMR, SULT4A1, CCDC78, CCDC151, FJX1, SLC23A3, CTNNA3, TEX101, BLK, ZMYND10, CH25H, CSRNP3, TP63, SLAMF6, ATP2A3, CCR7, TMEM154, KLHL32, DHRS9, FGD2, AGR2, UBASH3A, ARNT2, OR12D3, ACAP1, CXCR5, SLC44A4 |
| GSE55550 | 168 | GREM2, PDE6A, CXCL3, ALDH1L1, COL22A1, LPO, UGT2A2, IFNE, RTP3, UPK1A, CCR8, F2RL2, ZIC5, LPAR3, AGR3, BANK1, AMY1C, SFTA2, PCSK1, CALB1, OGN, MAGEA2B, CYP2F1, C5AR1, PPEF1, KRT1, IRX4, TLR10, AADAC, KCNS1, VPREB1, APCDD1L, SHISA3, PLAT, FBN2, CARD18, CXCL2, MEX3A, SLITRK5, A2ML1, RPS4Y2, HOXC9, WNT2, ASPG, ADGRF1, GDPD3, GDF10, FUT3, IQCJSCHIP1, HSPB3, KRT26, SCIN, TSPAN8, MUCL1, CAVIN4, SMC1B, AICDA, NEFM, SLC13A4, OXGR1, COMP, IRX2, XAGE1A, CXCL1, HOXB13, BCAS1, CNTNAP2, CST1, DTX1, LRMP, HLF, SERPINA9, FCRL4, CXCL8, ECM1, SLC47A2, SNX31, SH2D1B, DLX4, MAGEA1, SALL4, STAP1, ANXA9, DLX6, SCHIP1, SFRP2, CAPN6, RPTN, C9ORF152, VCAN, MYZAP, HTR3B, KRT24, TMEM132C, PLA2G4D, LOX, MSLN, SULF1, BMP8A, ADH7, SPINK6, RGS20, UPK3B, KRT76, PAX1, ITGB6, UCN2, MYRIP, ELF5, FCER2, HOXB9, CSF3, GCNT3, ADAMTS4, UNC13C, RSPO1, MAGEA12, HAS2, SOX11, P2RY12, NOX4, S100A7, CYP2E1, FUT5, HOXC8, SPRR4, PRR15L, HEPACAM2, SOST, TDO2, MMRN1, TM4SF19, TCTEX1D2, CD79B, FCAMR, FUT7, HOXC6, LAMA1, TUBB3, SPINK8, CCL3, TM4SF19, LHX2, RPS4Y1, CRISP2, ART4, GPR18, GP2, KHDRBS2, GABRA3, SCG5, FCRL1, TNFRSF12A, SERPINA5, GPM6A, FOSL1, SLC16A1, COL8A1, SEMA3C, RERGL, HTRA4, ACKR3, GAPT, HAPLN1, BNC1, SPOCD1, LHX1, SPOCK1, SERPINB11 |
| TGGA | 228 | RCN3, C16orf74, ITGA5, PDPN, CASQ1, TK1, MYO1B, HSPB6, ADAM12, GSTA1, COL5A3, LDB3, CHRDL1, IDO1, COL4A2, LUM, TPX2, HMGCS2, NID1, CGNL1, PEBP4, HOXD11, HOMER3, NETO2, IGF2BP2, SERPINH1, CD276, CXCL13, LY6K, PLIN4, BST2, ATP1A2, PLEK2, MYOM1, BPIFA1, CDC25B, IFI6, AGRN, TCN1, LOXL2, ZIC2, DYNAP, CCN4, BIRC5, PLK1, ACKR1, DNAH17, MB, UBE2C, CPXM1, MYL3, DMBT1, SPNS2, PLA2G2A, HOXD10, FSCN1, FOXM1, LGALS1, CCNB2, TNNT3, BPIFB2, P3H2, FNDC5, GPRIN1, C1QTNF6, KLHL41, HOXC10, AZGP1, MUC7, TTYH3, GPD1, ULBP2, TF, CHST11, MYBPC1, TNNC2, BARX2, GABRP, ALG1L, CXCL9, ALDH1A1, TCAP, E2F1, CTSV, APOBEC2, KLHDC7B, AEBP1, BMP1, CDC45, MYOT, TENM2, CDC6, CLEC3B, MYH7, MCM2, SOCS1, PKMYT1, ATP13A4, TNNC1, MYBL2, ADAMTS2, HOXD13, SNX10, MYH2, AURKA, CEACAM7, PADI2, COX7A1, SH2D2A, MUC15, PRH1, IFI27, ENO3, EMP1, COL4A6, CA3, ATP2A1, AURKB, PRH2, DEPTOR, C6orf58, MUC5B, RSAD2, COL6A2, NDRG2, ANO1, SLN, FST, ADH1B, FKBP10, KIF4A, DES, THY1, COX6A2, TNNI2, FSTL3, CASP14, PPP1R1A, KRT36, FAM107A, RORC, GPR176, ENO2, KIF2C, GPX3, NRAP, LMOD2, PPP1R3C, AIM2, ACTA1, ITGA6, SPINK7, COL6A1, CLDN10, CERCAM, MT1A, PI16, LRRC15, CENPA, SPARC, STRIT1, SCNN1B, MYL1, CEACAM1, PMEPA1, MELK, MAGEA3, SMPX, EPSTI1, CKMT2, PYGM, GBP5, KLK12, CDC20, SCGB3A1, PRAME, MYOZ1, HSPB7, CTHRC1, IL36G, APOL1, TGFB1, S100A1, GAST, XIRP2, CHST2, HOXB7, MYL2, RBP1, TMEM132A, TGFBI, PLOD3, NELL2, PRR4, CKM, MYLPF, LAMA3, BGN, SH2D5, GPD1L, DUSP9, OASL, SFRP1, PRB3, PDK4, SELENBP1, ACTN2, TRIP13, TYMP, APOC1, TMPRSS2, CDCA5, CYP4B1, IFI44, SLC2A1, HMGA2, KRT23, ISG15, ARTN, ADAMTS12, EYA2, ST6GALNAC1, IFIT3, LAMP5, HOPX, AMTN, EEF1A2, CDCA8 |
