# Supplementary material for: Aberrant Methylation and Immune Microenvironment Are Associated With Overexpressed Fibronectin 1: A Diagnostic and Prognostic Target in Head and Neck Squamous Cell Carcinoma
Source: Front Mol Biosci. 2021 Oct 20;8:753563. doi: 10.3389/fmolb.2021.753563 (PMC8563786; doi:10.3389/fmolb.2021.753563)
Supplement: Supplementary file 2 [file Table2.DOCX]

**Supplementary Table 2.** The differentially expressed genes with significant prognostic capacity in HNSCC

| Genes | HR | Low 95% CI | Hight 95% CI | *p* value |
| --- | --- | --- | --- | --- |
| FN1 | 1.333 | 1.018 | 1.746 | 0.037 |
| PLAU | 1.533 | 1.170 | 1.170 | 0.002 |
| FAM3D | 0.750 | 0.573 | 0.573 | 0.036 |

HR, hazard ratio; CI, confidence interval.
