# Supplementary material for: Aberrant Methylation and Immune Microenvironment Are Associated With Overexpressed Fibronectin 1: A Diagnostic and Prognostic Target in Head and Neck Squamous Cell Carcinoma
Source: Front Mol Biosci. 2021 Oct 20;8:753563. doi: 10.3389/fmolb.2021.753563 (PMC8563786; doi:10.3389/fmolb.2021.753563)
Supplement: Supplementary file 3 [file Table3.DOCX]

**Supplementary Table 3.** The details of patients diagnosed with HNSCC in our clinic

| Clinical variable | Number (n=20) |
| --- | --- |
| Gender |  |
| Male | 13 (65.0%) |
| Female | 7 (53.0%) |
| Age |  |
| ＜60 | 8 (40.0%) |
| ≥60 | 12 (60.0%) |
| Tumor size (mm) | 27.70±14.71 |
| Tumor stage |  |
| T1 | 4 (20.0%) |
| T2 | 5 (25.0%) |
| T4 | 3 (15.0%) |
| T4 | 8 (40.0%) |
| Lymph metastasis |  |
| N0 | 12 (60.0%) |
| N1 | 5 (25.0%) |
| N2 | 3 (15.0%) |
| TNM classification |  |
| Ⅰ | 4 (20.0%) |
| Ⅱ | 4 (20.0%) |
| Ⅲ | 2 (10.0%) |
| Ⅳ | 10 (50.0%) |
| Smoking status |  |
| Yes | 5 (25.0%) |
| No | 15 (75.0%) |
