# Supplementary material for: Aberrant Methylation and Immune Microenvironment Are Associated With Overexpressed Fibronectin 1: A Diagnostic and Prognostic Target in Head and Neck Squamous Cell Carcinoma
Source: Front Mol Biosci. 2021 Oct 20;8:753563. doi: 10.3389/fmolb.2021.753563 (PMC8563786; doi:10.3389/fmolb.2021.753563)
Supplement: Supplementary file 4 [file Table4.DOCX]

**Supplementary Table 4.** The details of TNM classification in HNSCC

| TNM classification | T | N | M |
| --- | --- | --- | --- |
| I | T1 | N0 | M0 |
| II | T2 | N0 | M0 |
| III | T3 | N0 | M0 |
| III | T1, T2, T3 | N1 | M0 |
| IVA | T4a | N0, N1 | M0 |
| IVA | T1, T2, T3, T4a | N2 | M0 |
| IVB | Any T | N3 | M0 |
| IVB | T4b | Any N | M0 |
| IVC | Any T | Any N | M1 |

T, primary tumor size and site; N, regional lymph node involvement; M, presence or otherwise of distant metastatic spread.
